# Peyton’s four-step approach for teaching complex spinal manipulation techniques – a prospective randomized trial

**DOI:** 10.1186/s12909-016-0804-0

**Published:** 2016-11-03

**Authors:** Gertraud Gradl-Dietsch, Cavan Lübke, Klemens Horst, Melanie Simon, Ali Modabber, Tolga T. Sönmez, Ralf Münker, Sven Nebelung, Matthias Knobe

**Affiliations:** 1 Department of Orthopaedic Trauma, Medical Faculty, RWTH Aachen University, 30 Pauwelsstreet, 52074 Aachen, Germany; 2 Medical Faculty, RWTH Aachen University, 30 Pauwelsstreet, 52074 Aachen, Germany; 3 Department of Oral and Maxillofacial and Plastic Facial Surgery, Medical Faculty, RWTH Aachen University, 30 Pauwelsstreet, 52074 Aachen, Germany; 4 Department of Orthopaedics and Trauma Surgery, Bethlehem Hospital Stolberg, 5 Steinfeldstreet, 52222 Stolberg, (Rheinland) Germany; 5 Department of Radiology, Medical Faculty, RWTH Aachen University, 30 Pauwelsstreet, 52074 Aachen, Germany

**Keywords:** Medical education, Spinal manipulation, Instructional method, Gender differences, Peyton’s four-step approach

## Abstract

**Background:**

The objectives of this prospective randomized trial were to assess the impact of Peyton’s four-step approach on the acquisition of complex psychomotor skills and to examine the influence of gender on learning outcomes.

**Methods:**

We randomly assigned 95 third to fifth year medical students to an intervention group which received instructions according to Peyton (PG) or a control group, which received conventional teaching (CG). Both groups attended four sessions on the principles of manual therapy and specific manipulative and diagnostic techniques for the spine. We assessed differences in theoretical knowledge (multiple choice (MC) exam) and practical skills (Objective Structured Practical Examination (OSPE)) with respect to type of intervention and gender. Participants took a second OSPE 6 months after completion of the course.

**Results:**

There were no differences between groups with respect to the MC exam. Students in the PG group scored significantly higher in the OSPE. Gender had no additional impact. Results of the second OSPE showed a significant decline in competency regardless of gender and type of intervention.

**Conclusions:**

Peyton’s approach is superior to standard instruction for teaching complex spinal manipulation skills regardless of gender. Skills retention was equally low for both techniques.

## Background

Manual therapy including manipulation, mobilization, and traction is frequently used in the treatment of musculoskeletal disorders. However, there remain many unanswered questions with respect to training modalities and associated levels of competence.

The need of a medical expert with a strong didactical background to transfer these complex psychomotor skills was shown [1]. The mastery of complex psychomotor skills is a prerequisite for chiropractic treatments. Students typically acquire these skills through observation of their teachers demonstrating specific procedures and through practice on fellow students [2]. There is growing evidence that motor learning principles such as mental practice [3], augmented feedback [4] or different training schedules [5] can promote skills acquisition. However, a recent review found insufficient evidence to make definitive recommendations for the use of different motor learning principles in skills training [6]. Rodney Peyton’s four-step approach has been proven to be effective in skills lab training of technical skills [7, 8]. The approach comprises four clearly defined instructional steps [9]:

- Step 1 – “Demonstrate”: The trainer demonstrates the skill at a normal pace and without additional comments.
- Step 2 – “Talk the trainee through”: The trainer demonstrates the respective skill while describing each procedural substep in detail.
- Step 3 – “Trainee talks trainer through”: The trainer performs the skill for a third time, based on the substeps described to him by the trainee.
- Step 4 – “Trainee does”: The trainee performs the skill on his/her own.

Potential benefits of the technique include the combination of several learning theories.

Especially step three, when the trainee instructs the trainer, seems to be a key to student learning. The student first has to reflect upon Steps 1 and 2 (Think) before instructing the trainer (Share). Think-Share allows the student time to organize their thoughts before actively articulating their thoughts [10]. In addition, the cognitive process called self-explanation seems to facilitate the integration of new knowledge into existing knowledge [11].

Medicine used to be a predominantly male occupation but today, woman account for half of all medical students in the USA and outnumber men in several European countries [12]. Gender and associated gender roles are reported to have an impact on learning and skills acquisition [13] and an influence on specialty preferences [14]. With rising numbers of women in medical school these differences warrant further evaluation in order to accommodate the educational needs of both genders.

This investigation evaluated (1) whether Peyton’s four-step approach is superior to conventional instruction for teaching complex psychomotor skills to medical students, (2) whether effectiveness of the approach is related to gender, (3) how the teaching strategy is perceived by trainees and (4) whether skills are maintained over time.

We hypothesized that Peyton’s four-step approach would be effective and well accepted by trainees regardless of gender and would enhance skills retention over time.

## Methods

### Study design

This was a single-center prospective randomized trial. Institutional Review Board approval was granted before initiation of this study, and strict confidentiality guidelines were followed (Local Ethics Committee Reference Number EK 178/09).

### Randomization

We randomly assigned course participants (simple computerized random numbers) to an intervention group which received instructions according to Peyton’s four-step approach (PG) or a control group, which received conventional teaching (CG).

### Participant selection

**Fig. 1:**
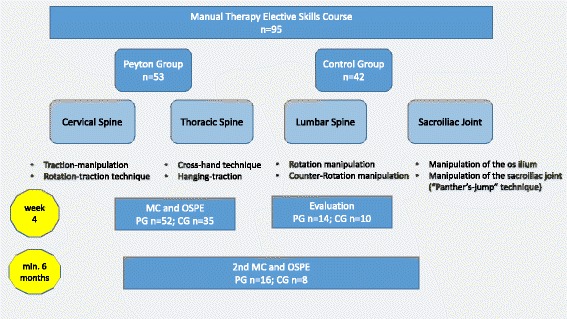
Detailed course schedule and flow of participants

Based on previous literature on the effect of Peyton’s four-step approach an effect size of 0.7 was expected. A standard power calculation (two tailed *t*-test, power = 0.8 and a = 0.05) indicated that a sample size of 34 participants is needed in each group to demonstrate this effect size [7]. Eligible participants were all medical students that took the elective Manual Therapy Course. Participants provided informed consent for the use of their results in this study at the time of enrollment. A total of 87 students completed both the course and assessment. The detailed schedule is depicted in Fig. 1. All students were recruited at one single university between October 2012 and October 2013.

### Course concept

**Fig. 2:**
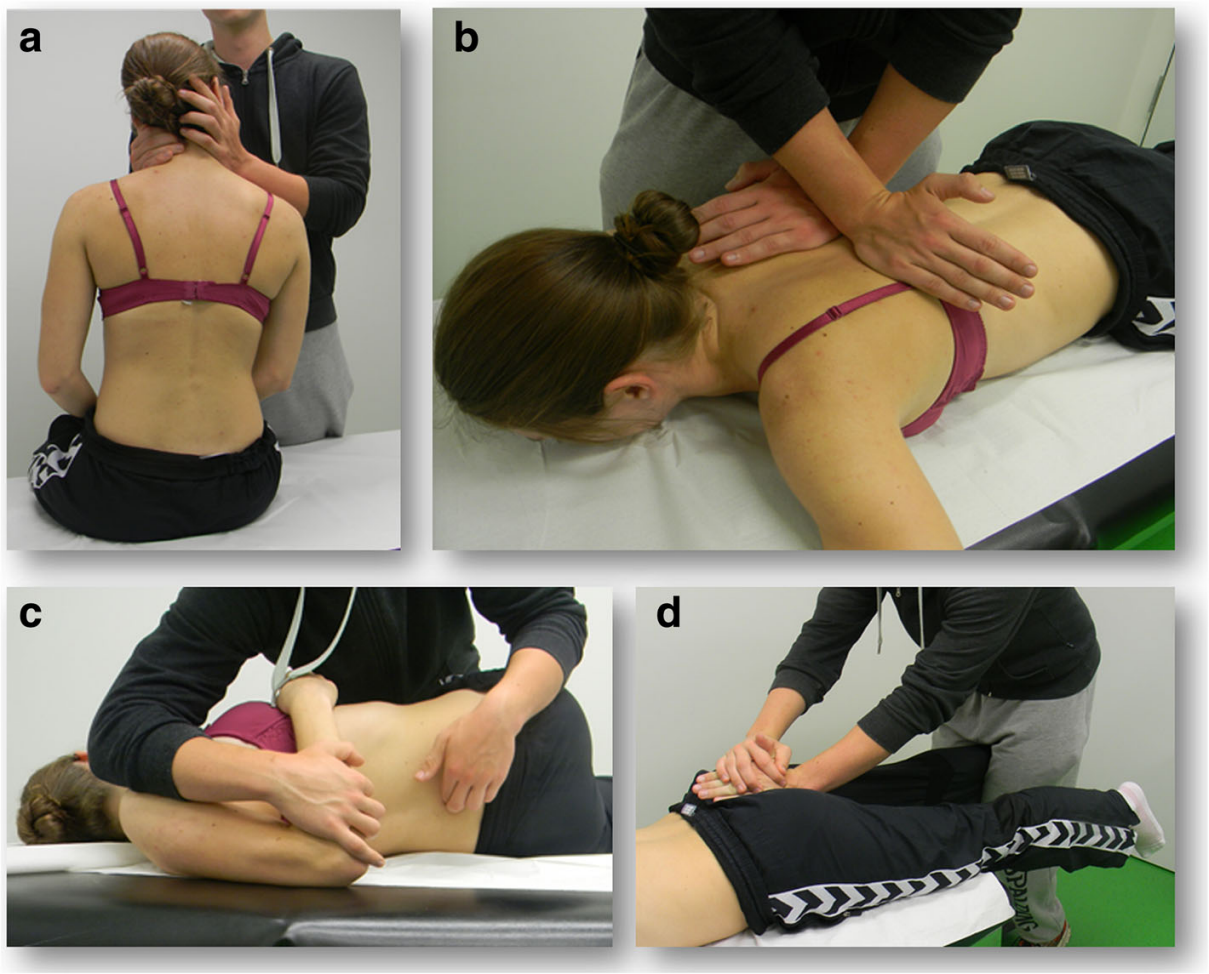
Students practising the **a**) Rotation-traction-technique for the cervical spine **b**) Cross-hand-technique for the thoracic spine **c**) Manipulation of the lumbar spine **d**) Manipulation of the sacroiliac joint (Panther’s jump technique)

We offered an elective skills course for third to fifth year medical students. In four, 120 min long sessions, we covered principles of manual therapy and specific manipulative and diagnostic techniques for the spine, including the sacroiliac joint. The 30 min theoretical introduction to each session included indications and contraindications for manual therapy, differences between mobilisation and manipulation, diagnostics, patient positioning, hand placement, specific anatomical contact, preload, thrust phases and the direction of force. In the training part (90 min), students practised the 3-step-diagnosis of the spine and techniques for each segment of the spine and the sacroiliac joint (Fig. 2a-d).

### Teachers

Students were taught by two board-certified orthopaedic consultants that both held a certificate in manual therapy and had at least 7 years of experience and one student tutor per group. Student tutors received thorough instructions for their respective teaching session prior to the study. Instructors were not blinded to the study design but taught only one method (Peyton or standard instruction) to avoid reciprocal interference.

### Training according to Peyton (Peyton group)

Steps 1 and 2 were performed for the whole group. Steps 3 and 4 were performed by all students individually for each skill with a teacher to student ratio of about 1:1. Afterwards, students received feedback about their performance.

### Standard instruction (conventional group)

Standard instruction comprised demonstration of practical skills by the teacher accompanied by explanations and time for students to ask questions. Students then practised the skills on each other receiving assistance and feedback by the teacher and student tutor.

### Assessment

At the end of the course, students took a 10 items multiple choice (MC) exam on the principles of manual therapy as taught in the theoretical part of the course. Skills acquisition was assessed in an Objective Structured Practical Examination (OSPE) [15, 16]. In order to assure objectivity and exact evaluation, the exams were videotaped. Three independent observers who were blind to the aim of the study and its design assessed students’ performance using a 100 item binary checklist (Table 1).

**Table 1:** OSPE Checklist

Name:		Rater #	
Student ID:			
Exam Date:			
Item	1. Cervical Spinal	Correct	Incorrect
	a) Three-Step-Diagnosis		
1	Examiner faces the patient		
2	Locates point of irritation (IP) (one finger’s breadth lateral of the spinous process)		
3	Consults the patient for pain		
	Segmental hypomobility		
4	Palpates the spinous processes C4-C6		
5	Checks cervical rotation		
6	Checks cervical flexion		
	Response of the IP to movement		
7	Palpates IP		
8	Checks cervical rotation		
9	Consults the patient for increase/decrease in pain intensity and change in consistency of the IP during movement		
	b) Rotation-traction-technique		
	*Positioning of the patient*		
10	Upright position, adequate seat height		
11	Examiner stands beside the patient		
	*Hand placement*		
12	Proximal phalanx of the thumb level to zygoma		
13	Forefinger yoke		
14	Other hand immobilizes inferior border of vertebral arch		
15	15°-degree tilt of the head to farside of the examiner		
16	15°-degree rotation to uninvolved side		
17	Builds up pre-tension (traction/rotation)		
	*Test traction*		
18	Further rotation of the neck		
19	Consults the patient for increase in pain intensity, other symptoms, dizziness		
20	Returns to pre-tension		
	*Manipulation*		
21	Rotational impulse to uninvolved side		
22	Manipulation in expiration		
	2. Thoracic spine	Correct	Incorrect
	a) Three-Step-Diagnosis		
23	Patient is in prone position		
24	Locates point of irritation (IP) (one finger’s breadth lateral of the spinous process)		
25	Consults the patient for pain		
	*Segmental hypomobility*		
26	Palpates the spinous processes (three adjacent vertebrae)		
27	Checks rotation (lifts arm)		
28	Checks flexion		
	*Response of the IP to movement*		
29	Palpates IP		
30	Checks rotation (lifts arm)		
31	Consults the patient for increase/decrease in pain intensity and change in consistency of the IP during movement		
	b) Cross-hand-technique		
	*Positioning of the patient*		
32	Patient is in prone position		
33	Positions him/herself on rotation-sensitive side		
	*Hand placement*		
34	Places hypothenar eminence of left hand over posterior transverse process of dysfunctional segment		
35	Fingers pointing cranially		
36	Places hypothenar eminence of right hand over opposite side transverse process approximately one segment below dysfunctional segment		
37	Fingers pointing laterally		
38	Builds up pre-tension		
	*Test traction*		
39	Puts more pressure on transverse process		
40	Consults the patient for increase in pain intensity, other symptoms, dizziness		
41	Returns to pre-tension		
	*Manipulation*		
42	Rotational impulse to uninvolved side		
43	Manipulation in expiration		
	3. Lumbar spine	Correct	Incorrect
	a) Three-Step-Diagnosis		
44	Locates point of irritation (IP) (one finger’s breadth lateral of the spinous process)		
45	Consults the patient for pain		
	*Segmental hypomobility*		
46	Palpates the spinous processes (three adjacent vertebrae)		
47	Checks rotation		
48	Checks flexion		
	*Response of the IP to movement*		
49	Patient is in prone position		
50	Palpates IP		
51	Checks rotation (lifts pelvis/shoulder on one side)		
52	Consults the patient for increase/decrease in pain intensity and change in consistency of the IP during movement		
	b) Counter-rotation manipulation		
53	Patient lies on their side		
54	Rotation-sensitive side up		
55	Patient is positioned on the edge of the exam table		
56	hip and knee of upper leg is flexed (90°)		
57	Examiner’s thigh secures tibial head of the patient		
58	Counter-rotation of the spine		
	*Hand placement*		
59	Index and Ringfinger guide the Middlefinger		
60	Places finger on the spinous process of the segment to be assessed		
61	Places arm on Os ilium		
62	Aligns arm with patient’s back (bridging)		
63	Builds up pre-tension		
	*Test traction*		
64	Applies traction		
65	Consults the patient for increase in pain intensity, other symptoms, dizziness		
66	Returns to pre-tension		
	*Manipulation*		
67	Rotational impulse to uninvolved side		
68	Manipulation in expiration		
	4. Sacroiliac joint	Correct	Incorrect
	a) Three-Step-Diagnosis		
69	Patient is in prone position		
70	Locates point of irritation (IP)		
71	three finger’s breadth lateral of the posterior superior iliac spine (PSIS)		
72	four finger’s breadth caudal of the iliac crest		
	*Segmental hypomobility*		
73	Patient stands upright		
74	Examiner is positioned behind the patient		
75	Places left thumb on left ASIS		
76	Places right thumb on right ASIS		
77	Asks patient to bend slowly forward		
78	Monitors PSIS downward motion on affected side		
	*Response of the IP to movement*		
79	Patient is in prone position		
80	Examiner is positioned behind the patient		
81	Palpates IP		
82	Checks cranialisation		
83	Checks caudalisation		
84	Checks ventralisation		
85	Checks dorsalisation		
86	Consults the patient for increase/decrease in pain intensity and change in consistency of the IP during movement		
	b) Panther’s jump technique		
87	Patient is in prone position		
88	Patients’ legs hang over the edge of the table		
89	Examiner is positioned at the foot of the table		
	*Hand placement*		
90	Affected leg is fixed between examiner’s lower thighs		
91	Uses ulnar edge of hand		
92	Places hand from a caudal direction on the affected side of the sacrum		
93	Places the other hand on top of the first		
	Builds up pre-tension		
94	→ Applying traction by carefully moving backwards		
95	→ Applying tangential force on the sacrum		
	*Test traction*		
96	Applies traction		
97	Consults the patient for increase in pain intensity, other symptoms, dizziness		
98	Returns to pre-tension		
	*Manipulation*		
99	Short thrust to uninvolved side		
100	Manipulation in expiration		
		Result	

### Evaluation

Participants were asked to evaluate the course using a paper-based 38 items survey. Questions focused on the quality of the individual course units (6-point grading scale; 1 = very good, 6 = insufficient) competence of the lecturer, teaching strategy, the quality and organisation of the lessons and the increase in skills and knowledge (5-point Likert scale; 1 = fully agree, 5 = strongly disagree).

### Assessment of skills retention

Participants were asked to take a second OSPE, identical to the first, at least 6 months after completion of the course. Again, the exams were videotaped and assessed by three independent observers. A total of 24 participants (Peyton Group: 9 women and 7 men, Control Group: 7 women and 1 men) were available for the second exam.

### Statistics

Descriptive statistics were computed for variables of interest. Chi Square or Fisher’s exact test was used to assess differences for categorical variables. We performed multivariate analysis to assess the relationship between exam scores and evaluation results as the dependent variable and gender, and type of intervention as predictors. We used repeated measures ANOVA to assess differences in retest results between groups. Significance level of statistical tests was set at *p* < 0.05. We used intra-class correlation (ICC, two-way mixed, average measures, absolute agreement) to assess interobserver reliability. An ICC value >0.7 was regarded as satisfactory [17]. The statistical analyses were performed using SPSS (version 22.0, IBM, USA).

## Results

### Study population

Table 2 summarizes the demographic characteristics of the participants. There were no significant differences between groups regarding gender or age (Table 2).

**Table 2:** Demographic data

		Peyton Group	Control Group	*p*
Gender (*n*)	women	31	22	1
	men	21	13	
Age* (years)		22 (18–32)	22 (20–35)	0.9

*Values are presented as median and range

### Assessment

Using Pillai’s trace, there was a significant effect of type of intervention on the results of the practical exam (*V* = 0.35, *F*(10,74) = 4.1; *p* < 0.001; *d* = 0.66).

Gender had no significant effect on outcome in multivariate analysis (*V* = 0.18, *F*(10,74) = 1.6; *p* = 0.13) (Table 3).

**Table 3:** Exam results according to type of intervention and gender (Between subject factors - Univariate ANOVAs)

	Peyton Group		Control Group		Between Subject Factor Intervention		Between Subject Factor Gender	
	Women	Men	Women	Men	*F*(1,83)	*p*	*F*(1,83)	*p*
OSPE total score	57 ± 12.3	61.9 ± 12.4	54.7 ± 13.7	45.2 ± 20.3	8.9	0.004*	0.5	0.5
OSPE diagnostic part	22.5 ± 7.1	24.8 ± 5.6	24.3 ± 8.4	21.1 ± 9.5	0.3	0.6	0.1	0.8
OSPE therapeutic part	34.5 ± 8.1	36.6 ± 9.8	30.4 ± 8.1	24.1 ± 11.7	16.4	<0.001*	1.1	0.3
Cervical spine Diagnosis	5.5 ± 1.4	5.9 ± 1.6	5.8 ± 1.9	6.2 ± 1.9	0.6	0.5	0.9	0.3
Cervical spine Therapy	5.4 ± 4.1	6.3 ± 4.2	5.4 ± 2.9	4.7 ± 3.6	0.7	0.4	0.02	0.9
Thoracic spine Diagnosis	5.1 ± 1.6	5.8 ± 1.2	5.5 ± 1.6	5.2 ± 2.1	0.1	0.8	0.4	0.5
Thoracic spine Therapy	8.6 ± 2.3	9.8 ± 2.1	8.2 ± 2.4	7.2 ± 3.3	7.4	0.008*	0.02	0.9
Lumbar spine Diagnosis	3 ± 2.4	4.3 ± 2	3.4 ± 2.7	3.3 ± 2.7	0.3	0.6	1.2	0.3
Lumbar spine Therapy	10.3 ± 3.7	10.7 ± 3.9	8.7 ± 3.4	6.6 ± 4.9	11.3	0.001*	0.9	0.3
Sacroiliac joint Diagnosis	8.9 ± 3.9	8.9 ± 3.4	9.6 ± 4.4	6.4 ± 4.4	0.9	0.3	3.1	0.08
Sacroiliac joint Therapy	10.2 ± 1.5	9.8 ± 3.1	8.2 ± 2.7	5.6 ± 4.5	24.8	<0.001*	5.3	0.02*
Multiple choice exam	6.8 ± 1.6	7.4 ± 1.5	6.8 ± 2.1	5.9 ± 1.8	3.7	0.06	0.2	0.7

Values are presented as mean ± standard deviation, *indicating significance

Using Pillai’s trace there was a significant decline in knowledge and procedural skills regardless of type of intervention (Table 4). Due to the uneven distribution of women and men, we did not assess the impact of gender on the exam results.

**Table 4:** Results of the second DOPS exam (Repeated measures analysis compared to first DOPS)

	Peyton Group	Control Group	F	*p*
OSPE total score	42.8 ± 14	34.6 ± 26.9	(1,18) 27	<0.001*
OSPE diagnostic part	20.1 ± 7.1	19.1 ± 10.9	(1,18) 4.4	0.04*
OSPE therapeutic part	22.8 ± 8.9	15.5 ± 16.7	(1,18) 40.1	<0.001*
Cervical spine Diagnosis	4.7 ± 1.2	4.6 ± 1.9	(1,17) 3.9	0.06
Cervical spine Therapy	3.8 ± 2.9	2.4 ± 3.9	(1,17) 11.3	0.004*
Thoracic spine Diagnosis	4.6 ± 1.6	4.2 ± 2.8	(1,17) 1.1	0.29
Thoracic spine Therapy	6.7 ± 2.8	5.7 ± 3.6	(1,17) 25.4	<0.001*
Lumbar spine Diagnosis	3.8 ± 2.1	4.2 ± 2.4	(1,18) 1.5	0.2
Lumbar spine Therapy	5 ± 4.4	3.7 ± 6.1	(1,18) 34.1	<0.001*
Sacroiliac joint Diagnosis	7.1 ± 3.1	6.4 ± 4.8	(1,18) 11.8	0.003*
Sacroiliac joint Therapy	7.3 ± 3	4.5 ± 4.2	(1,18) 15.2	0.001*

Values are presented as mean ± standard deviation. *indicating significance

### Evaluation

Complete questionnaires were available from 23 participants, equivalent to a response rate of 26 %. Using Pillai’s trace, there was no significant effect of neither type of intervention (*V* = 0.98, *F*(1,19) = 2.7; *p* = 0.4) nor gender (*V* = 0.95, *F*(1,19) = 1.1; *p* = 0.7) on the evaluation results (Table 5).

**Table 5:** Evaluation

Evaluation Item	Peyton Group		Control Group		Between Subject Factor Intervention		Between Subject Factor Gender	
	Women	Men	Women	Men	*F*(1,19)	*p*	*F*(1,19)	*p*
Cervical spine and thoracic spine (6-point grading scale)								
Indications/contraindications	2.4 ± 1.3	2.1 ± 0.4	1.8 ± 0.4	2	1.35	0.26	0.02	0.9
Mobilisation/Manipulation	2.3 ± 0.9	1.9 ± 0.7	2.4 ± 1.1	2.25 ± 0.5	0.5	0.5	0.6	0.4
3-step-diagnosis	1.9 ± 0.9	1.7 ± 0.7	1.8 ± 1.3	2	0.1	0.8	0.01	0.9
Hand placement	2.1 ± 1.1	2.1 ± 0.4	2.6 ± 0.9	2.25 ± 0.5	0.7	0.4	0.3	0.6
3-step-diagnosis cervical&thoracic spine	1.9 ± 0.9	2.1 ± 0.7	2.4 ± 1.1	2	0.3	0.6	0.03	0.9
Traction-manipulation of the cervical spine	2.3 ± 0.5	2.1 ± 0.7	2.2 ± 0.4	2.3 ± 0.5	<0.001	0.9	0.04	0.8
Rotation-traction technique, cervical spine	2 ± 0.6	2 ± 0.6	2.4 ± 1.1	2	0.4	0.5	0.4	0.5
Cross-hand technique, thoracic spine	2 ± 0.8	1.9 ± 0.7	2.2 ± 0.8	2	0.3	0.6	0.3	0.6
Cervical spine and thoracic spine (5-point Likert scale)								
The instructor was knowledgeable about the subject	1.1 ± 0.4	1.7 ± 0.5	1.2 ± 0.4	1.3 ± 0.5	1.1	0.3	2.6	0.1
The instructor-learner interaction was positive	1.4 ± 0.5	1.6 ± 0.8	1.6 ± 0.5	1.5 ± 0.6	0.03	0.9	0.01	0.9
The instructor answered my questions to my satisfaction	2 ± 1	2.1 ± 0.7	2.8 ± 0.8	1.5 ± 0.6	0.1	0.8	2.7	0.1
I enjoyed the course	1.6 ± 0.8	1.6 ± 0.5	1.6 ± 0.5	1.5 ± 0.6	0.01	0.9	0.03	0.9
In this course I learned a great deal	1.9 ± 0.9	1.9 ± 1.1	3.2 ± 0.8	2.25 ± 0.5	5.1	0.04*	1.5	0.2
I feel confident to apply the practiced techniques to real patients	3.3 ± 1.3	3.4 ± 1.5	4.2 ± 0.8	3.8 ± 1.5	1.2	0.3	0.07	0.8
The course provided an appropriate balance between instruction and practice	2.1 ± 1	2.3 ± 0.8	2 ± 0.7	2.3 ± 0.5	0.06	0.8	0.3	0.6
I would have rather been trained in the other group	4.3 ± 0.8	5	2.8 ± 1.3	4.5 ± 1	7.7	0.01*	11.4	0.003*
It would require more training to become proficient	1.6 ± 0.8	2.4 ± 1.6	1.4 ± 0.5	2.5 ± 1.7	0.01	0.9	3.3	0.08
The size of the class was appropriate	2.7 ± 1.1	2.4 ± 0.9	2.8 ± 1.6	1.8 ± 0.5	0.4	0.5	1.9	0.2
Lumbar spine and sacroiliac joint (6-point grading scale)								
Indications/contraindications	2.6 ± 0.5	2.3 ± 0.5	2.2 ± 0.4	2.5 ± 0.6	0.1	0.7	0.001	0.9
Mobilisation/manipulation	2 ± 0.8	2 ± 0.6	2.8 ± 0.8	3.3 ± 0.5	11.4	0.003*	0.5	0.5
3-step-diagnosis	2 ± 0.8	2 ± 0.6	2 ± 1.2	2.5 ± 1.3	0.4	0.5	0.4	0.5
Hand placement	2 ± 0.6	2.1 ± 0.4	2.8 ± 0.8	2.8 ± 0.9	6.1	0.02*	0.02	0.8
3-step-diagnosis lumbar spine	1.9 ± 0.7	2 ± 0.8	2.2 ± 1.1	2.3 ± 0.5	0.7	0.4	0.1	0.8
Rotation manipulation, lumbar spine	2.1 ± 1.1	2 ± 0.6	2.2 ± 1.1	2.5 ± 0.6	0.5	0.5	0.04	0.8
Counter-Rotation manipulation, lumbar spine	2.6 ± 0.8	2.6 ± 0.8	2.4 ± 0.9	2.8 ± 0.9	9.8	0.9	0.2	0.6
3-step-diagnosis sacroiliac joint	2 ± 0.8	2.1 ± 0.7	3 ± 1.2	2.8 ± 0.5	4.9	0.04*	0.02	0.8
Manipulation of the os ilium (sideways position)	2.6 ± 0.8	3 ± 1.2	2.8 ± 0.8	2.3 ± 0.9	0.4	0.5	0.02	0.9
“Panther’s-jump” technique	1.4 ± 0.5	1.9 ± 0.4	2 ± 0.7	2.5 ± 0.6	6.8	0.02*	3.9	0.06
Lumbar spine and sacroiliac joint (5-point Likert scale)								
The instructor was knowledgeable about the subject	1.4 ± 0.5	1.6 ± 0.5	1.4 ± 0.5	1.5 ± 0.6	0.05	0.83	0.3	0.6
The instructor-learner interaction was positive	1.3 ± 0.5	1.7 ± 0.8	1.8 ± 0.4	2 ± 0.8	2.2	0.16	1.3	0.3
The instructor answered my questions to my satisfaction	1.6 ± 0.9	2 ± 1	3 ± 1.2	2	2.9	0.1	0.5	0.5
I enjoyed the course	1.6 ± 0.8	1.4 ± 0.5	2 ± 1	1.8 ± 0.5	1.4	0.2	0.4	0.5
In this course I learned a great deal	2 ± 0.8	2.1 ± 1.3	3.4 ± 1.1	3 ± 1.4	5	0.04*	0.1	0.8
I feel confident to apply the practiced techniques to real patients	3.7 ± 0.8	3.4 ± 1.5	4.4 ± 0.5	3.5 ± 1.3	0.6	0.4	1.6	0.2
The course provided an appropriate balance between instruction and practice	1.9 ± 0.9	2.1 ± 0.7	2.6 ± 0.5	2.8 ± 0.9	4	0.05	0.4	0.5
I would have rather been trained in the other group	4.3 ± 0.9	5	2.8 ± 1.3	4 ± 1.4	8.8	0.008*	5.2	0.03*
It would require more training to become proficient	1.4 ± 0.5	2.3 ± 1.7	1.4 ± 0.5	1.5 ± 0.6	0.8	0.4	1.1	0.3
The size of the class was appropriate	2.1 ± 1.1	2.3 ± 0.9	3 ± 1	1.8 ± 0.5	0.2	0.7	1.9	0.2

Values are presented as mean ± standard deviation, *indicating significance

6-point grading scale, 1 = very good, 6 = insufficient; 5-point Likert scale, 1 = fully agree, 5 = strongly disagree

### Interrater reliability

A high degree of reliability was found between raters for all variables of interest (Table 6).

**Table 6:** Interrater Reliability

	Intra-class correlation (ICC)	Intra-class correlation (ICC) 2nd DOPS
OSPE total score	0.985 (0.975–0.991)	0.988 (0.971–0.995)
OSPE diagnostic part	0.98 (0.971–0.986)	0.978 (0.956–0.990)
OSPE therapeutic part	0.981 (0.970–0.988)	0.988 (0.972–0.995)
Cervical spine Diagnosis	0.926 (0.895–0.949)	0.943 (0.887–0.973)
Cervical spine Therapy	0.968 (0.955–0.978)	0.964 (0.929–0.983)
Thoracic spine Diagnosis	0.923 (0.891–0.947)	0.965 (0.931–0.984)
Thoracic spine Therapy	0.947 (0.925–0.963)	0.970 (0.936–0.986)
Lumbar spine Diagnosis	0.966 (0.952–0.977)	0.917 (0.821–0.963)
Lumbar spine Therapy	0.976 (0.965–0.984)	0.990 (0.981–0.996)
Sacroiliac joint Diagnosis	0.977 (0.967–0.984)	0.964 (0.929–0.983)
Sacroiliac joint Therapy	0.947 (0.913–0.967)	0.972 (0.942–0.987)

Values are presented as average measures with the 95 % Confidence interval in brackets

## Discussion

This prospective randomized trial investigated two different approaches for teaching complex spinal manual therapy techniques in an elective skills course. Theoretical instruction did not differ between groups and there were no differences between groups with respect to the results of the multiple choice exam. However, analysis of the videotaped practical exam revealed significant differences between instructional approaches. Students that received instructions according to Peyton’s four-step approach scored significantly higher in the overall score and especially in the more complex therapeutic parts. Although gender had no additional impact in multivariate analysis, univariate analysis suggests that men seemed to benefit more from this instructional approach than women. Participants were asked to take a second OSPE, identical to the first, at least 6 months after completion of the course and results showed a significant decline in knowledge and competency to perform techniques. Students in the Peyton Group again performed better than in the Control Group. However, with only a small number of students available, this difference did not reach significance. A recent randomized controlled trial investigating the differential learning outcomes of the separate steps of Peyton’s four-step approach identified Peyton’s Step 3 as the most crucial part of the technique [18]. Students that received Peyton’s Steps 1, 2, and 3 showed a significantly superior first independent performance of central venous catheter insertion using a manikin compared to students that received only steps 1 and 2. Results of an incidental free recall test 1 day after training showed similar outcomes. The significant decline in competency in both groups in our trial might be attributed to the far longer latency, at least 6 months compared to 1 day, between tests.

This decline in knowledge and skills might be attributed to the fact that spinal manipulation is not part of students’ daily routine and that they had no opportunity to apply their acquired knowledge and skills in the meantime.

Repeated training as well as periodic formative assessments might be possible solutions to the described skills and knowledge decay [19, 20]. Concrete changes to the course curriculum could involve a longitudinal, modular concept to promote skills retention.

Complex bimanual tasks of spinal manipulation require high levels of sensory and motor coordination and confidence and should be taught by experienced professionals [1]. Existing guidelines offer no indication as to the comprehensiveness of training necessary or for the standard of competence that should be attained. Motor tasks requiring whole body coordination are especially challenging because they depend on the coordination of trunk and limb movements [21]. Thus, an early implementation of training musculoskeletal examination and motor skill techniques during medical school could be highly beneficial, especially considering the fact that medical students do not feel adequately prepared in musculoskeletal medicine [22]. Several alternative teaching methods have been used for teaching spinal manipulation such as quantitative augmented feedback strategies or special manikin or simulator training [4, 23]. Interestingly, peer teaching, which has been proven to be effective for teaching technical skills, seems not to be beneficial [1].

Rodney Peyton’s four-step approach has been reported to be a useful strategy for teaching complex manual skills [7, 8] and results of our trial prove its effectiveness in spinal manipulation.

Given the complexity of Peyton’s four-step approach, one might assume that the length of time needed for instruction will be far greater than in the Control Group. With equal training hours, this should provide the Control Group with comparatively more time to practice and thus higher levels of competency. One possible explanation for the superiority of the Peyton Group might be the combination of motor imagery and skills performance as inherent in Peyton’s Step 3 [18, 24, 25]. This is supported by the results of a randomized controlled trial evaluating the impact of a cognitive training method on the performance of simulated laparoscopic cholecystectomy. Surgeons that received additional mental training outperformed both a group that received additional practical training as well as a control group and regarded mental training as a valuable tool in their education [26].

Evaluation results showed that students in the Peyton Group had the impression that they had learned a lot and students in the Control Group, especially women, reported that they would have rather been in the Peyton Group. A qualitative study used focus group discussions to find out what teaching skills helped students to acquire physical examination skills [27]. Students wanted teachers to demonstrate a skill step-by-step as opposed to showing the whole examination at once to prevent memory loss. Students also acknowledged the positive effects of demonstrating skills in front of the class, such as direct feedback [23].

Several reports suggest gender differences in learning and skills acquisition. Men tend to perform better in tasks requiring visuospatial abilities, have more confidence in their surgical abilities and take more risks [13, 28]. However, providing women with certain instructional approaches such as feedback and one-to-one training seems to eliminate these differences [28, 29]. This is reflected in the evaluation results, where significantly more women reported that they would have rather been in the Peyton Group. Results of a previous trial comparing peer-assisted learning to expert training of manual therapy revealed no gender differences with regard to theoretical or practical knowledge. Despite the fact that students in the expert group outperformed students in the peer group, women in the peer group rated the effectiveness of the teaching method as fairly good, found their teachers competent and enjoyed the course [1]. This might be attributed to a more positive teacher/trainee interaction and again emphasizes the importance of training programs that acknowledge the different needs of participants.

### Limitations

This was a single-center study. Results may differ in different organisational or didactical settings. Furthermore, we did not assess the level of any anatomical knowledge or skills competency concerning manipulation techniques acquired prior to the intervention. However, according to the curriculum, students had not received any spinal manipulation training on the musculoskeletal system prior to the study and students denied any such qualifications in the questionnaire. We could not control for autonomous self-study and students’ motivation which might have influenced the final test results. We do not see this as a threat to internal validity since selection bias was controlled by including a large number of participants and using methods of complete random sampling. The study guideline allowed students to miss one class during the entire course. Frequency and timing of absence had no significant influence on the final result. Results of the multiple choice exam did not differ between groups which might be attributable to the fact that theoretical teaching was identical for both groups and the fact that the number of MC questions might have been too low.

Results of the second OSPE should be interpreted with caution due to the small number of participants. In addition, the response rate to our survey was fairly low, creating potential non-response bias.

## Conclusions

Results of our trial suggest that Peyton’s four-step approach is superior to standard instruction for teaching complex spinal manipulation skills to medical students regardless of gender. The teaching concept is suitable for training even large groups and is well accepted by trainees. However, skills retention was equally low for both techniques.
